# Microbiome plasticity during the early life-stage of the angelshark (*Squatina squatina*) in Las Teresitas (Canary Islands)

**DOI:** 10.1371/journal.pone.0361287

**Published:** 2026-10-28

**Authors:** Tomas Bañeras, David Jiménez-Alvarado, Hector Toledo-Padilla, Laura Camps-Lahora, José Juan Castro, Ana Espino-Ruano, Airam Guerra-Marrero, Lorena Couce-Montero, Eva K.M. Meyers, Lluís Bañeras

**Affiliations:** 1 IU-ECOAQUA, Universidad de Las Palmas de Gran Canaria, Las Palmas, Spain; 2 Angel Shark Project: Canary Islands, Islas Canarias, Spain; 3 Group of Molecular Microbial Ecology, Institute of Aquatic Ecology, University of Girona, Girona, Spain; 4 LIB, Museum Koenig Bonn, Leibniz Institute for the Analysis of Biodiversity Change Bonn, Germany; 5 Universidad del Atlántico Medio, Las Palmas de Gran Canaria, Spain; University of Messina, ITALY

## Abstract

Shark microbiome studies offer acute insights into host health, immunity, and ecological interactions, yet remain poorly studied compared to other marine vertebrates. Shark-associated microbial communities, despite being species-specific, are influenced by environmental conditions and feeding ecology. In this study, we aimed to characterize shifts in the microbiome of the Critically Endangered angelshark (*Squatina squatina*) during the early stages of development and to assess how environmental factors contribute to the shaping of a specific microbiota. Samples from thirty-nine juvenile angelsharks were collected from Las Teresitas nursery area in the Canary Islands over one year. Skin, oral and cloacal microbiomes were analysed using 16S rRNA gene amplicon sequencing. The sizes of individuals ranged between 24 and 42 cm in length. The shark microbiome was clearly distinct from the surrounding environment, indicating a unique composition from early life stages. Dominant bacteria present in the shark microbiomes included *Vibrionaceae* (up to 96%), *Pseudoalteromonas* (up to 63%), *Rhodobacteraceae* (up to 57%) and *Alteromonadaceae* (up to 20%). Seawater had a greater influence than sediment on the external skin microbiome, especially in younger individuals. This was not expected due to the burial behaviour of the species. Notably, the abundance of *Pseudoalteromonadaceae* (assessed by qPCR) was positively correlated with shark growth but was independent of the skin mucus. This suggests a protective role of this phylotype in younger individuals and may help in the adaptation to habitat conditions. This is the first comprehensive microbiome study in angelsharks, highlighting the importance of environmental preservation for microbial integrity and juvenile fitness.

## 1. Introduction

Specific shark bacterial communities have been described in several species [1–4]. Most studies agree that in the microbiome of sharks is a dynamic and adaptable system shaped by both host-associated and environmental factors. For instance, faecal and oral microbiomes are primarily influenced by internal factors such as host identity and diet, which help define a specific core set of bacteria [5]. In contrast, external skin microbiomes are strongly affected by environmental exposure (e.g. pH, salinity and temperature), generally leading to more complex and flexible microbial communities [5–8].

Host-microbe interactions often mirror host physiology, contribute to immune modulation, and limit pathogen colonization through competitive exclusion [9,10]. However, environmental disturbances can destabilize this equilibrium, increasing vulnerability to opportunistic pathogens and leading to dysbiosis [11–13]. Given that the microbiome can influence host health and stress response, its dynamics are of particular importance for endangered elasmobranchs such as the common angelshark. The common angelshark (*Squatina squatina*) is listed as Critically Endangered by the International Union for Conservation of Nature (IUCN) Red List of Threatened Species [14]. Detailed knowledge of its ecology, behaviour, and habitat requirements, particularly during the early life stages, remains limited but is essential for ensuring population sustainability [15]. The Canary Islands represent a unique stronghold for this species, yet populations there continue to face threats including habitat degradation, fishing pressure, and pollution [16,17].

The skin of elasmobranchs exhibits unique biophysical properties including dermal denticles (microscopic, tooth-like scales) that enhance hydrodynamic efficiency and largely influence bacterial colonization [18]. In fact, biomimetic shark skin surfaces have been employed to prevent biofilm formation [19]. Experimental evidence indicates that moderate skin surface roughness and structural complexity impact biofilm formation, and the coexistence of microbial species [18,20]. The epidermis of elasmobranchs is often covered by a thin, protein-rich mucus layer that adds selectivity to microbial colonization and helps shape a distinctive skin microbiome [1]. In addition, external skin microbial communities are continuously influenced by interactions with the surrounding seawater [1] and, in species that spend much of their time buried in sandy bottoms, by close contact with sediment.

The cloacal microbiome, in contrast to the skin, provides a useful proxy for assessing gut microbial composition [21]. In vertebrates, gastrointestinal microbes are closely associated with digestion, development, immune modulation, pathogen suppression, and overall health [22,23]. While most research on the gastrointestinal microbiome has focused on mammals, similar functional roles are presumed in fishes [24]. In teleost species, the composition of microbial communities is conditioned by a combination of environmental, physiological, and genetic factors, with diet likely playing a central role [9,25]. In addition, the gastrointestinal microbiome of elasmobranchs may also be influenced by their nitrogen metabolism, particularly the use of urea for protein synthesis and osmoregulation [25,26]. The oral microbiome of vertebrates has long attracted interest as a key interface between internal and external microbial communities, due to its role in oral diseases and broader systemic health [27]. In sharks, several studies have identified potentially pathogenic microorganisms in the oral microbiota, raising concerns about the infection risks from shark bite wounds [28,29]. Microbial diversity, richness, and composition in the oral cavity have been shown to vary across shark species, likely reflecting species-specific differences in diet and feeding behaviour [2].

The microbiome of juvenile angelsharks remains largely unexplored. Despite the growing recognition of the importance of host-associated microbial communities in elasmobranch health and ecology, their analysis in young individuals is technically challenging. This study provides the first characterization of the microbiome of juvenile angelsharks, constituting a relevant advancement in understanding early life microbial associations in this threatened species. To achieve this, juvenile individuals were sampled across different body sites (skin, cloaca, and oral cavity) over a one-year period within the Las Teresitas nursery area. We aimed to characterize microbiome composition and identify recurrent core taxa associated with *Squatina squatina* host microbiome. We also investigated the relationship between shark-associated microbiomes and environmental microbial communities (water and sediment) to evaluate potential influences on microbiome structure and their implications for host health. Additionally, we examined whether ontogenetic growth may contribute to microbiome plasticity and adaptation to changing environmental conditions.

## 2. Materials and methods

### 2.1. Study area

This study was conducted at Las Teresitas beach, Tenerife, Spain (28º 30’ 34.101’‘ N 16º 11’ 06.841’‘W) (Fig 1), an artificial beach where the original black volcanic sand was replaced in 1973 with sand imported from the Sahara Desert [30]. Las Teresitas is protected by a breakwater and features a shallow, sandy bottom reaching depths of approximately 10 meters. Given the spatial extent of the sampling area (71.000 m^2^), it was divided into three zones. At every campaign, the three zones were monitored consecutively over three nights from west (Zone 1) to east (Zone 3), being the central one (Zone 2) the less exposed to open water.

**Fig 1:**
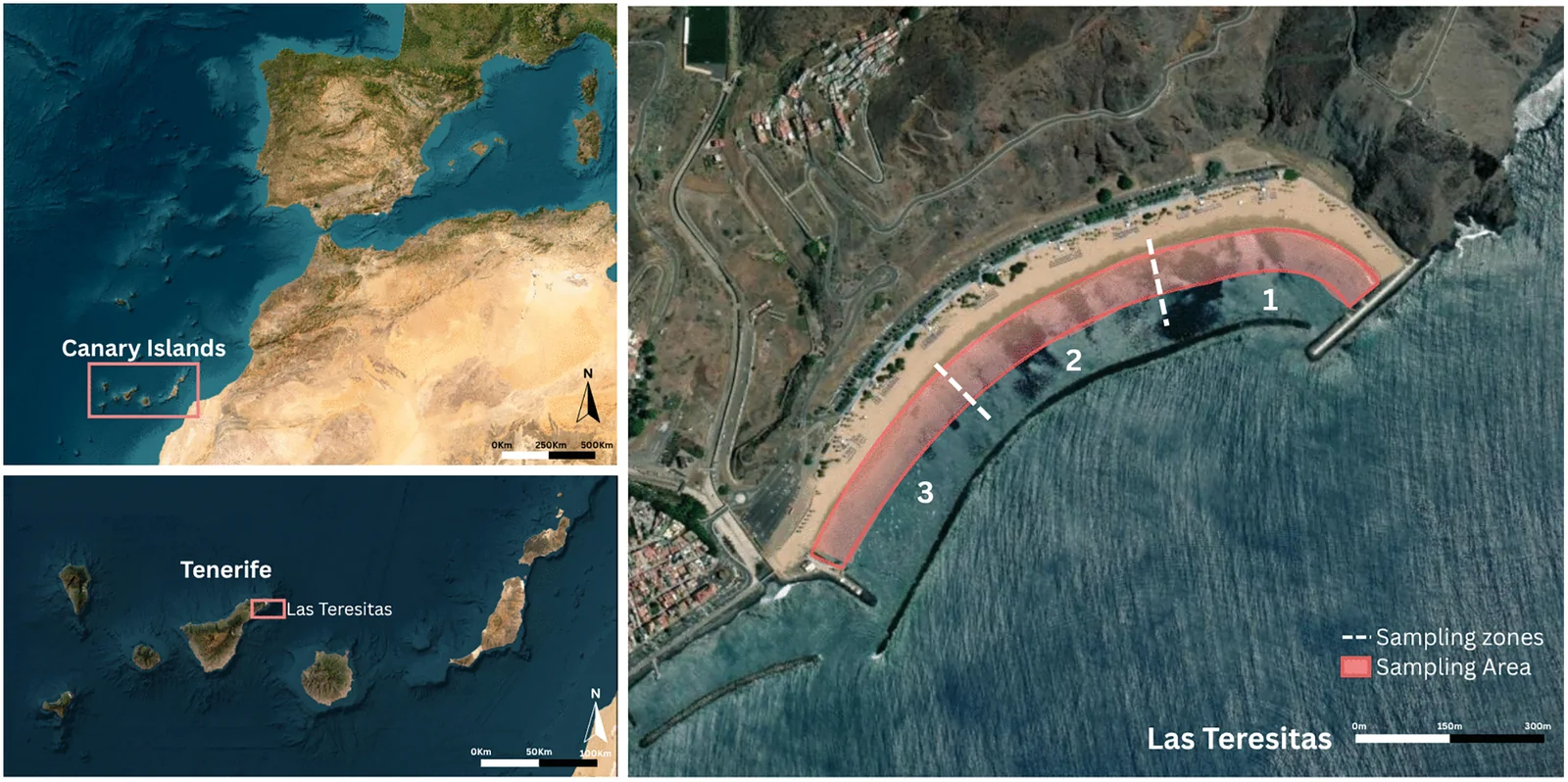
Las Teresitas, study area. Satellite images of the study area showing (a) the location of the Canary Islands in the eastern Atlantic Ocean, (b) the position of Las Teresitas on Tenerife Island, and (c) the spatial distribution of Las Teresitas beach, with the sampling area extension highlighted. Created from U.S. Geological Survey EarthExplorer imagery (ESRI World Imagery; accessed June 1, 2026, via https://earthexplorer.usgs.gov) and stylized using Adobe Illustrator 2024.

### 2.2. Sampling

Sampling campaigns were conducted between December 2023 and December 2024 by the Angel Shark Project (ASP), following the procedure described in The Tagging and Tracking Toolkit for Angelsharks [31]. Juvenile angelsharks were localized visually and collected in areas of water with maximum depths of 2.5 metres. Individuals were placed in a sea water filled plastic tray and manipulated in a land station. A total of thirty-nine juvenile angelsharks were sampled to assess their microbiomes at different body parts (skin, mouth and cloaca). Each juvenile was identified with a Passive Integrated Transponder (PIT) tags providing a long term and unique identification number that can be read electronically, allowing mark-recapture studies and long-term assessment of site fidelity, growth and survival. Among all captures, four individuals were sampled twice, and one individual was sampled three times, making a total of 45 samples. Sampling took place in December 2023 (n = 18), March 2024 (n = 1), June 2024 (n = 24), and December 2024 (n = 2) (S1 Table). Recaptured angelsharks were defined as individuals that had been tagged during a previous sampling campaign and were subsequently captured again in a later campaign.

Total length and weight of all specimens were recorded. In addition, during June 2024 the presence of mucus on the skin of *S. squatina* individuals was recorded. For this, a categorical classification was used based on visual inspection and manual handling of each shark. Individuals with tacky or resistant skin texture were classified as having low (or no) mucus, whereas those with smooth and slippery skin were categorized as having a higher mucus content.

Sterile swabs (APTACA SPA, Canelli, Italy) were used to collect the microbiome of the skin, the oral cavity, and the cloaca as follows: (i) skin swabs were taken from the dorsal region between the head and first dorsal fin by thoroughly swabbing over a 10 cm² area; (ii) cloacal samples were collected by gently inserting and rotating. Samples were only obtained if the cloaca cavity was bigger than 1.5 cm in length to prevent injury to animals; and (iii) oral samples were obtained by swabbing the upper and lower gingiva after carefully opening the shark’s mouth. All manipulations were performed wearing ethanol rinsed, air dried, dust free gloves. All swabs were stored on ice immediately after sampling and subsequently frozen at −20 °C. All handling procedures were conducted following established protocols and ethical guidelines provided by the Angel Shark Project (https://angelsharkproject.com/). Fieldwork was carried out under permits issued by the Ministry of Environment and Ecological Transition of Spain and the Government of the Canary Islands (ref. SGBTM/BDM/AUTSPP/03/2023 and SGBTM/BDM/AUTSPP/05/2024). Procedures were reviewed and approved by the Animal Ethics Committees of the Zoological Society of London (AQS 2, 2022). The manipulation of animals lasted for a maximum of two minutes and individuals were immediately released afterwards.

Water samples (n = 6) of the three different zones were collected using sterile Anaclin bottles at 0.5 m depth. Once in the lab (one hour driving distance from the sampling site), water was filtered through 0.22 μm pore size polycarbonate membrane filters (Merck Millipore, Milan, Italy). Filters were stored at −20 °C until further processing. Sea water temperature and dissolved oxygen was monitored using a multiparameter probe (YSI, ProDSS Multiparameter Digital Water Quality Meter). Sediment samples (n = 9) were also collected by hand at 0.8 m depth using sterile 50 mL falcon tubes. The overlying water was decanted immediately after collection. Retained sediment samples were frozen to −20ºC until DNA extraction. Due to field campaign logistics and restrictions on site, no environmental samples were collected during December 2024. Sampling was only directed to increasing the number of recaptured individuals.

### 2.3. DNA extraction and sequencing

Genomic DNA was extracted using the DNeasy PowerSoil Kit (QIAGEN). The manufacturer’s instructions were followed with minor adjustments depending on the sample matrix. For swabs, the cotton tip was aseptically cut using sterile scissors and directly inserted into the lysis tube containing beads and lysis buffer. For sediment samples, 0.6 grams of sediment were added to the lysis tube. Polycarbonate membrane filters were cut into small pieces using sterile forceps and scissors and added to the lysis tube. In all cases, extraction was carried out according to the standard protocol with a final elution step of 75 μL. DNA extracts were quantified using the Qubit dsDNA High Sensitivity Assay Kit (Invitrogen) and stored at −20 °C until further processing.

All DNA extracts were assessed for amplification using the universal bacterial/archaeal primer pair 515F and 806R targeting the V3-V4 hypervariable region of the 16S rRNA gene. Amplification and library preparation followed the standardized protocols established by the Earth Microbiome Project (EMP) [32]. High-throughput sequencing was performed using the Illumina MiSeq platform with a 2 × 150 bp paired-end read format (PE150). Library preparation and sequencing steps were outsourced and carried out at the SGI-IKER core facilities at the University of the Basque Country (UPV/EHU) in Bizkaia, Spain. The resulting raw sequencing data were used for downstream bioinformatic analyses of microbial community composition.

### 2.4. Quantitative determinations by qPCR

Quantitative PCR (qPCR) was used to estimate the absolute abundance of total *Bacteria*, and specifically the abundance of *Pseudoalteromonadaceae* and *Vibrio* spp. across samples. The bacterial 16S rRNA gene was targeted using the primer pair 341F–534R [33]. *Pseudoalteromonadaceae* were targeted with the specific Psalt815R primer combined with the 341F [34], whereas the abundance of *Vibrio* sp. was assessed using the 567F/680R primer pair according to Thompson [35].

qPCR reactions were performed in a Roche LightCycler 96 (Roche). qPCR reactions contained: 0.25 µM of each primer, 1X LightCycler® 480 SYBR Green I Master, and PCR grade water to a final volume of 18 µL/reaction. 2 µL of extracted DNA samples or dilutions were used in all cases to a final volume of 20 µL. Primer-specific annealing temperatures were set at 60 °C for Bacteria, 62°C for *Pseudoalteromonadaceae*, 58ºC *for Vibrio sp..* Apart from these differences, PCR cycles consisted of an initial denaturing step (95º C for 5 minutes), 40 cycles consisting of denaturation (95º C for 5 minutes), annealing (primer specific temperatures for 20 seconds) and extension steps (72ºC for 30 seconds). Fluorescence was recorded at the end of the extension phase. A melting curve analysis was performed at the end of each run to confirm amplification specificity. Standard curves were obtained by serial dilutions (10^8^ to 10^2^ copies/µL) of synthetic double-stranded DNA fragments for the three markers (gBlocks, Thermofisher). Amplification efficiencies were 89.8% for 16S rRNA gene, 84.7% for *Pseudoalteromonadaceae*, and 92,5% for *Vibrio sp.* Limits of quantification (LOQ) were set to 1.2 x 103 gene copies/µL for 16S rRNA gene, to 2.4 x 102 gene copies/µL for *Pseudoaltermonadaceae,* and to 5.7 x 101 gene copies/µL for *Vibrio sp*. LOQ was used for statistics when genes were not detected above respective values. In all cases, including standards and samples, three technical replicates were used.

### 2.5. Statistical analysis

All statistical analyses were conducted in R Statistical Software v4.4.2 [36], using phyloseq package. Figures were generated in R in the ggplot package and stylized with Adobe Illustrator (2024). Descriptive statistics (mean, standard deviation, minimum, and maximum) were calculated for the morphometric variables. Differences in morphometric measurements were assessed using analysis of variance (ANOVA), after confirming the assumptions of normality (Shapiro–Wilk test) and homogeneity of variances (Levene’s test). Statistical significance was assessed at α = 0.05.

Raw sequencing data were quality filtered, trimmed, dereplicated, merged and clustered into amplicon sequence variants (ASVs) using DADA2 v1.26 [37]. Briefly, reads were truncated at 240 (For) and 160 (Rev) bp with a maximum expected error (maxEE) of 2. No Ns were allowed in reads. ASVs were assigned per sample independently (pooling FALSE), reads merged and chimeras removed using the consensus method. Taxonomy was assigned according to Silva v138.2 database (www.arb-silva.de). The relative abundance of ASVs per sample was calculated using the phyloseq packages in R [38]. Amplicon sequence variants (ASVs) unassigned at the phylum level and samples containing fewer than 1,000 reads were excluded from the dataset, excluded samples were 45480DEC24S and 45543DEC23M. In total, 16,666 unique ASVs were obtained. To address unequal sequencing depth while retaining the maximum number of samples, the dataset was rarefied to 6,000 reads per sample. This option was chosen in opposition to a compositional transformation due to the large differences in the number of reads per sample in the data set [2],429–108,699). The similarity between the results outcome (rarefied vs non rarefied) was tested with a NMDS approach using the two datasets and a procrustes analysis of the sample distribution, revealing a very high concordance (symmetric Procrustes correlation = 0.9889; Procrustes sum of squares, m12² = 0.02208; 999 permutations, p = 0.001; S1 Fig). Taxonomic assignments were done using the Silva 138.1 database (www.arb-silva.de) at a minimum bootstrap level of 80%. Raw sequences obtained in this study have been submitted to the GenBank database under the SRA project accession number PRJNA1474969.

Core microbial communities were determined using the rarefied dataset according to Shade and Hadelsmann [1]. The contribution of bacterial families to community dissimilarity between shark body sites and environmental compartments was assessed through SIMPER analysis. Alpha diversity metrics, including the Shannon’s diversity index and richness (Number of observed ASVs), were estimated using phyloseq package. Normality and homogeneity of variances assumptions were not met for alpha diversity indices, therefore differences among body sites, sampling zones and dates, and the presence of skin mucus were tested using the Kruskal–Wallis test. If factors involved more than two levels (e.g., body site), post-hoc pairwise comparisons were performed using Dunn’s test with Benjamini–Hochberg false discovery rate (FDR) correction. Correlations between alpha diversity indices and morphometric measurements were evaluated using linear models.

Beta diversity among samples was estimated using Bray–Curtis dissimilarities, and samples were grouped according to a non-metric multidimensional scaling (NMDS) analysis. Differences according to body sites, sampling zones and dates, and the presence of skin mucus, were assessed using ANOSIM tests. To determine the effect of the environment (water and sediment) on the angelshark microbiome inverse Bray–Curtis distances were calculated on a pair-wise basis and correlated (Spearman’s rank correlation) to body length and weight.

Absolute or relative abundances (to the total 16S rRNA genes) of *Pseudoalteromonadaceae* and *Vibrio* were correlated to angelshark morphometric measurements using linear models.

## 3. Results

### 3.1. *Squatina squatina* bacterial community composition

Studied angelsharks had an average length of 30.19 ± 4.88 cm and an average weight of 272.37 ± 129.10 g (S2 Table). The sampling zone did not show a significant impact on shark length or weight (ANOVA, df = 2, p > 0.1) but significant differences were observed according to date, being the individuals sampled in December (n = 20) larger than those sampled in June (n = 24) (ANOVA, df = 2, p < 0.05). No seasonal differences were observed for water temperature or dissolved oxygen concentration.

Sequencing of 16S rRNA genes achieved higher accuracy results for all samples except for 45480DEC24S and 45543DEC23M, which were excluded from downstream analyses for having a low number of reads (< 1,000) (S3 Table). The 16S rRNA amplicon data produced 16,666 unique ASVs in the whole data set, of which 8,152 (48.9F%) were found exclusively in the shark microbiome (S2 Fig), suggesting a specific shark microbiome. The number of unique shared ASVs between *S. squatina* body sites and sediment samples was larger (1,167, 7%), when compared to water (123, 0.7%). A total of 5,246 (31.5%) ASVs were found uniquely in skin. Skin and sediment samples shared 985 ASVs (6%).

Skin-associated microbiomes of *S. squatina* were dominated by members of *Enterobacterales* (39.7% average relative abundance), followed by *Pseudomonadales* (10.3%), and *Rhodobacterales* (7.6%) (S3 Fig). In oral (mouth) and cloacal samples, the community was predominantly composed of *Enterobacterales* (61% and 62%, respectively) and *Pseudomonadales* (9% and 8,5%). In contrast, sediment and water bacterial communities showed a greater diversity, with a higher proportion of low-abundance groups at the Order level. Water samples were primarily composed of *Flavobacteriales* (24.9%), *Rhodobacterales* (18%), and *Enterobacterales* (17%), while sediment samples harbored relatively low proportions of dominant taxa, being *Flavobacteriales*, *Pseudomonadales*, and *Rhodobacterales* the most abundant, each of them contributing less than 5% to the total community. Archaea (mainly *Nitrosopumilaceae*) were only found at low relative abundances (6%) in sediment samples but were hardly detected in the shark microbiome and water (<1%).

At the family level, the most abundant taxa associated with angelsharks included *Vibrionaceae* (23.7%) and *Pseudoalteromonadaceae* (22.9%) (Fig 2, S4 Fig). *Pseudoalteromonas, Alteromonas, Vibrio* and *Staphylococcus* appeared as the most prevalent members in shark core communities, with small differences in their relative abundances among body-parts (S5 Fig). Interestingly, none of the four genera, except *Pseudoalteromonas s*p. were defined as a core member of the sediment or water microbial communities. Environmental water samples, by contrast, were enriched in *Rhodobacteraceae* (18.4%) and *Cryomorphaceae* (16.9%). Sediment samples showed a rather homogeneous microbiome composition with little changes according to date of sampling or sampled area. In contrast, water microbiome composition was more heterogenous. Samples collected during December 23 (WATERDEC23R1 and WATERDEC23R3) exhibited a completely different microbiome composition being *Alteromonadaceae* unexpectedly higher in those samples

**Fig 2:**
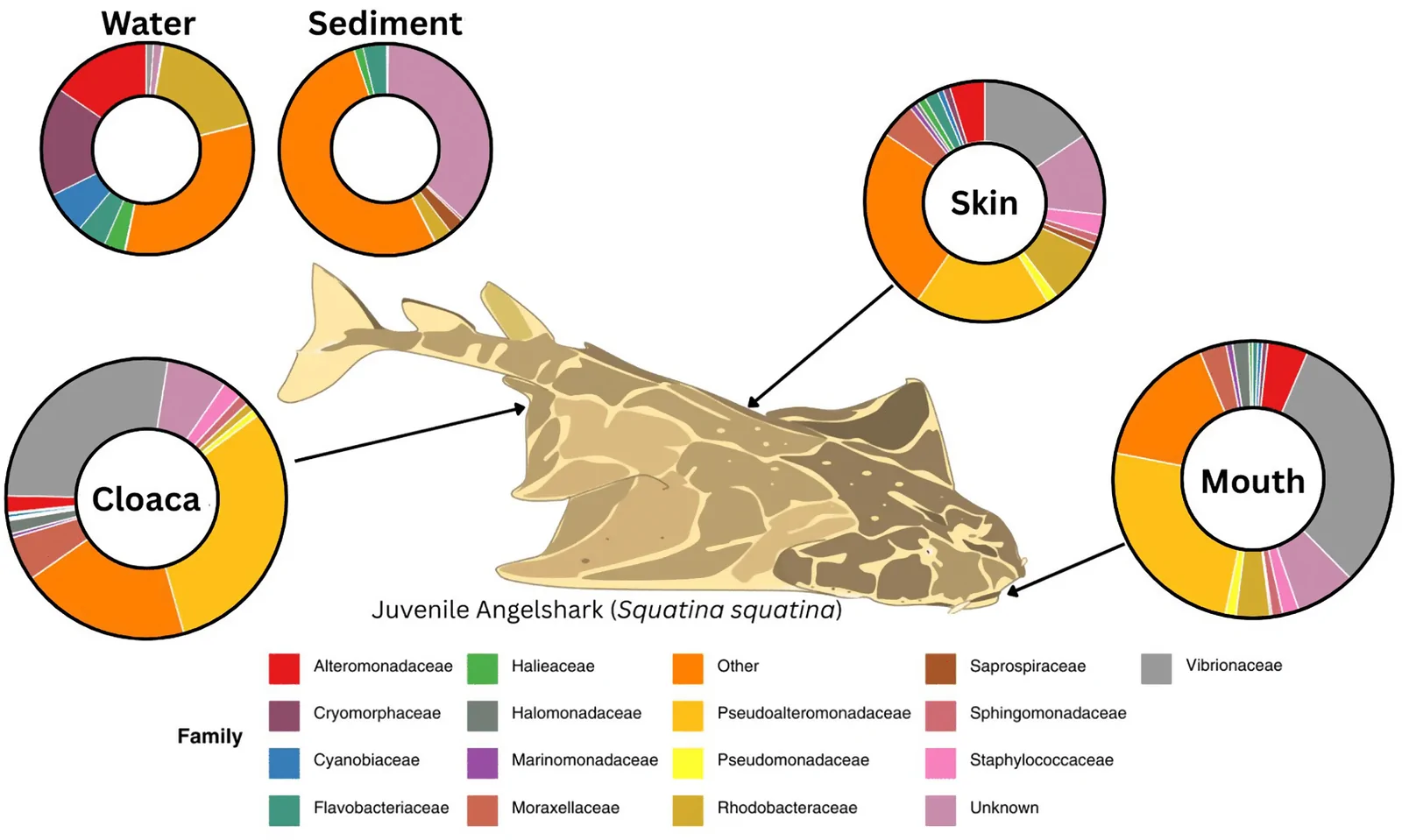
Family-level bacterial community structure in water, sediment, and body sites of juvenile angelsharks (*Squatina squatina).* Donut charts showing the most relatively abundant bacterial groups (Family level) in water, sediment and juvenile angelsharks (*Squatina squatina*) body parts (skin, mouth and cloaca) collected in Las Teresitas beach, Tenerife. Values are averages of all samples within the five groups. Unknown: Sequences not classified at the Family levels. Others: Families occurring at relative abundances lower than 1% or less than 20% prevalence.

A SIMPER analysis suggested that differences in bacterial community between, shark body sites and environmental compartments were driven by specific taxa, mainly by *Vibrionaceae* and Pseudoalteromonadaceae (S6 Fig), with lower influence of *Rhodobacteraceae*, *Moraxellaceae* and *Alteromonadaceae*. Despite being the most relevant groups, the relative abundance of *Vibrionaceae* and *Pseudoalteromonadaceae* showed a high variability among shark related samples, changing from values above 70% to almost undetectable (S4 Fig). The abundance of *Vibrio* sp. and *Pseudoalteromonadaceae* was confirmed by qPCR. The abundance of the two groups was rather heterogeneous and varied from 3.9 × 10^2^ to 3.72 × 10^5^ copies/cm^2^ and from 1.82 × 10³ to 3.03 × 10^6^ copies/cm^2^ of skin for *Vibrio sp*. and *Pseudoalteromonadaceae*, respectively. Relative abundances (as per total bacteria 16S rRNA gene abundance) ranged from 81% to nearly undetectable for *Pseudoalteromonadaceae*, and from 0.01 to 21% for *Vibrio* sp.

### 3.2. *Squatina squatina* microbiome alpha and beta diversity analyses

Alpha diversity indices reinforced the idea of a specific microbiome in sharks compared to either water or sediment. Significantly higher Shannon’s diversity and Richness index were found in sediment samples (Observed richness, 1297 ± 163 ASVs; Shannon’s index, 6.53 ± 0.142) compared to water and angelshark microbiomes (Kruskal–Wallis test Benjamini-Hochberg, χ² = 36.458, df = 4, p < 0.001, Fig 3). In contrast, alpha indices of water-associated microbiomes did not differ significantly from those related to the *S. squatina* body parts in most cases (Kruskal-Wallis Benjamini-Hochberg, χ² = 52.7973, df = 4, p > 0.01, Fig 3). Among shark-associated samples, the lowest alpha diversity was observed in the cloaca (Observed richness, 129 ± 111 ASVs; Shannon’s, 2.77 ± 1.15) and the highest in the skin (Observed richness, 319 ± 194 ASVs; Shannon’s, 3.82 ± 1.14). *S. squatina* microbiome clusters partially overlapped according to an NMDS ordination (Fig 3) but differed completely from sediment and water microbial communities. Multivariate dispersion was low among groups (betadisper, 999 permutation, p = 0.05), with water samples being less variable than shark-associated and sediment samples. Shark-associated samples showed the highest dispersion (p = 0.003), driven by greater variability in skin microbiomes compared to cloaca and mouth (Tukey’s HSD, p < 0.05). Differences among groups were assessed with a pairwise ANOSIM test, confirming completely distinct community structures between shark-associated samples and sediment microbiomes (R = 0.957 for skin, R = 0.997 for mouth, and R = 1.000 for cloaca). Similar but slightly lower R values were estimated in comparison with water samples (R = 0.801, 0.930, and 0.959, for skin, mouth and cloaca, respectively). Among angelshark body parts, no significant differences were observed (cloaca vs. mouth (R = 0.07), cloaca vs. skin (R = 0.05), and mouth vs. skin (R = 0.05)).

**Fig 3:**
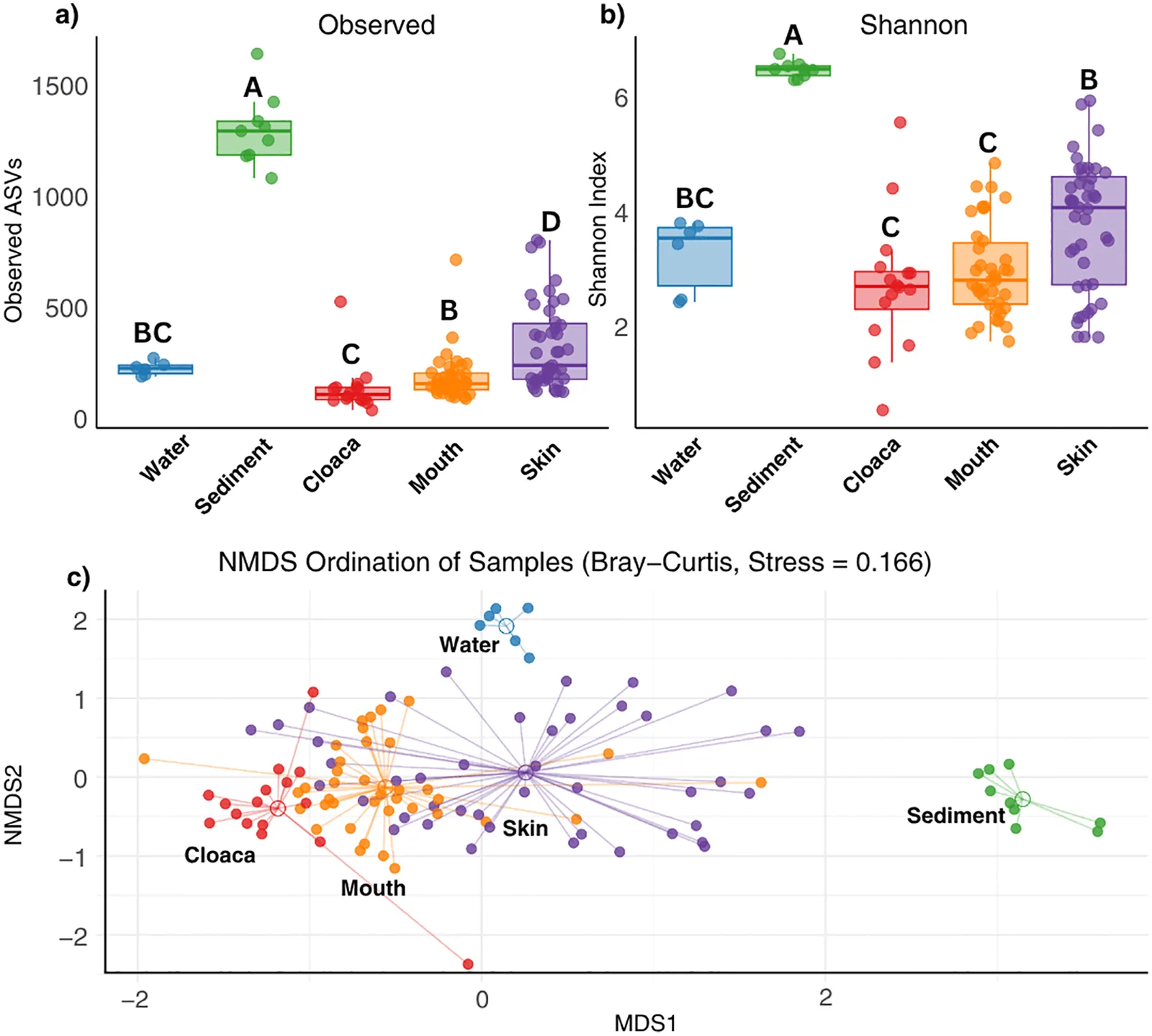
Comparison of bacterial diversity and community composition among environmental samples and body sites of juvenile angelsharks (*Squatina squatina*). (a) Box and whisker plots of bacterial alpha diversity indicators, Richness (number of observed ASVs) and Shannon’s diversity (H′) for the five sample types in *Squatina squatina*. Upperccase letters indicate differences among groups (Dunn’s post hoc test). b) Non-metric multidimensional scaling (NMDS) ordination of bacterial communities detected in Sediment, Water and different body sites of juvenile angelsharks from Las Teresitas beach, Tenerife. Individual points for all samples are shown. Vectors show distances to the centroid (empty dot) for every sample group.

### 3.3. Growth associated microbiome shifts

Alpha diversity indices were related to morphometric variation using linear regression models. Significant negative relationships were detected between skin microbiome richness and diversity and body length, with both observed richness and Shannon diversity decreasing with increasing length (Observed richness–Length: LM, adjusted R² = 0.1417, df = 41, p = 0.0074; Shannon diversity–Length: LM, adjusted R² = 0.096, df = 41, p = 0.02; Fig 4). Similar patterns were observed with body weight (Observed richness–Weight: LM, adjusted R² = 0.1757, df = 41, p = 0.0030; Shannon diversity–Weight: LM, adjusted R² = 0.22, df = 41, p = 0.00115; S7 Fig). No significant relationships were observed for cloacal or oral microbiomes (S8 Fig).

**Fig 4:**
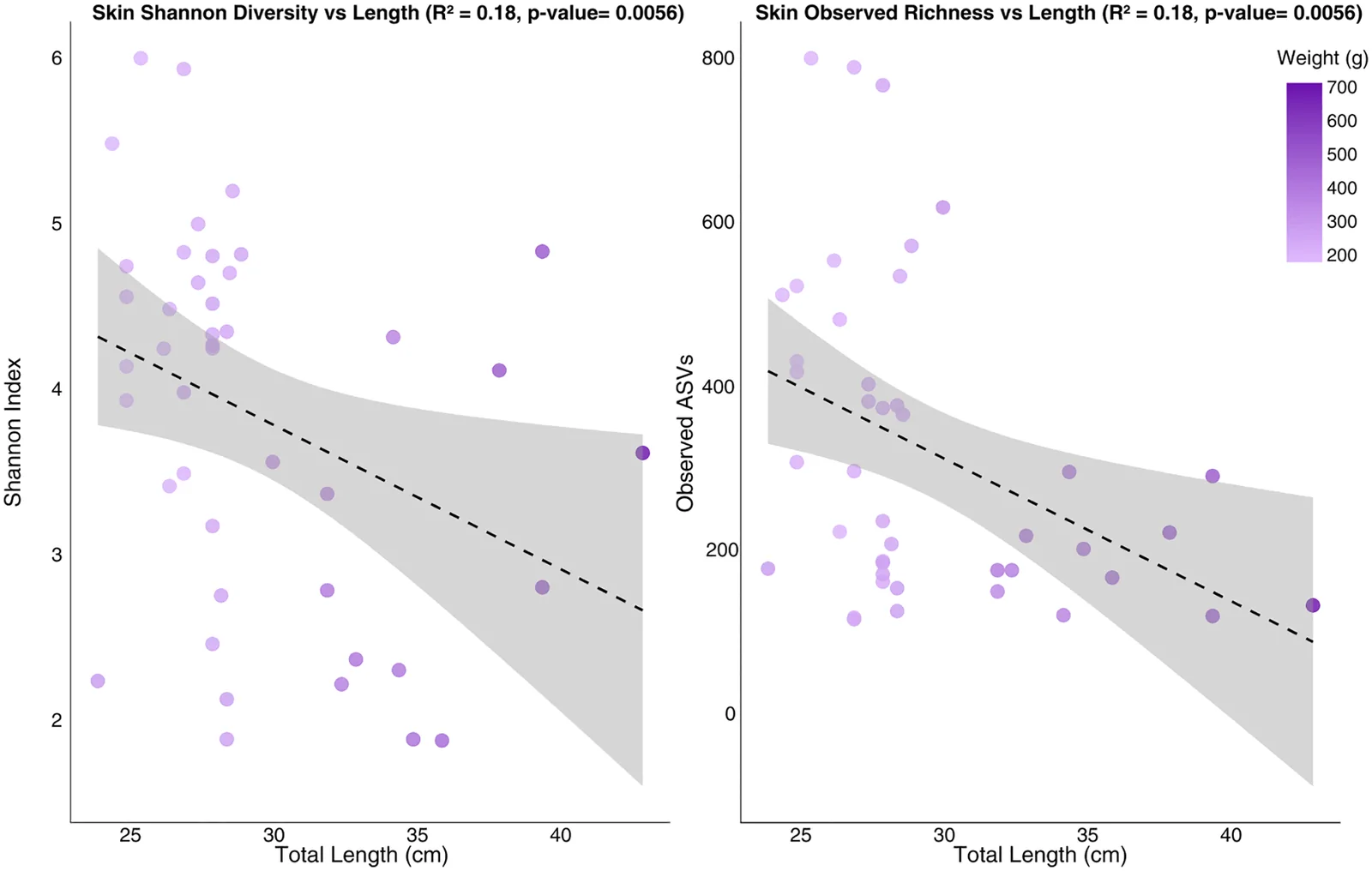
Relationship between skin bacterial diversity and body size in juvenile angelsharks (*Squatina squatina*). Scatter plots showing the correlation between Shannon diversity (left) and observed richness (right) index values in skin samples with *Squatina squatina* total length (cm). Colour gradient of dots represents weight differences being darker than heavier individuals. Dashed line and grey areas represent confidence interval (CI), 95% for the fitted regression line.

Abundance of Pseudoalteromonadaceae in skin samples was positively related to total shark length (LM on log_10_-transformed abundance: R² = 0.208, df = 43, p < 0.05; Fig 5). In contrast, the abundance of Vibrio sp. was not correlated with length despite a positive trend observed. The increasing trend of Pseudoalteromonadaceae according to shark size could not be fully confirmed with data obtained from the recaptured individuals (IDs 45480, 45513, 45462, and 45528, Fig 5). Whereas Pseudoalteromonadaceae increased according to size in 45513 (from June 2024 to December 2024) and 45480 (from December 2023 to June 2024), it decreased in 45562 and 45528 despite the increases in length of more than 5 cm. The abundance of Vibrio sp. showed a decreasing trend in most cases.

**Fig 5:**
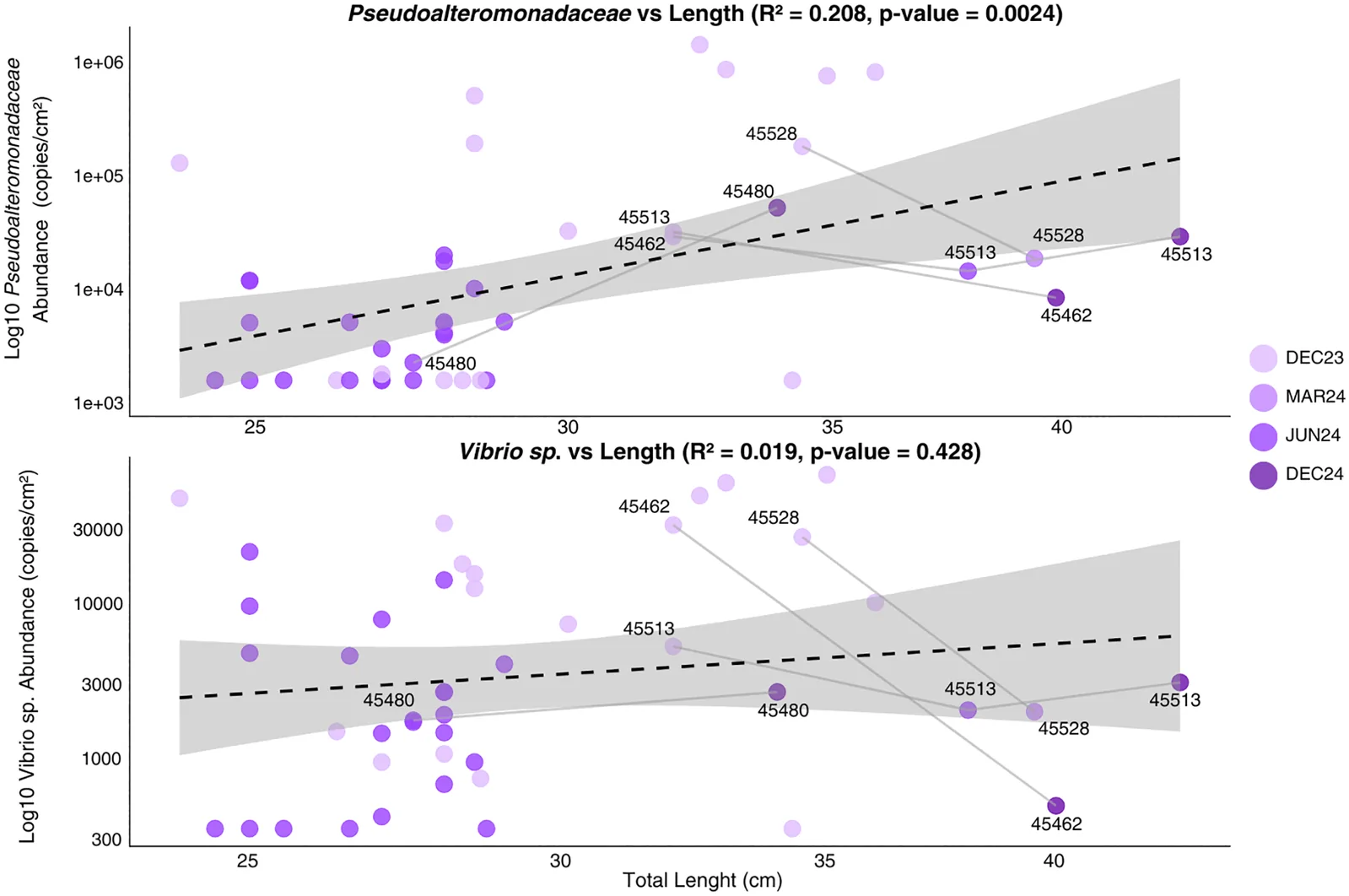
Correlation of *Pseudoalteromonaceae* and *Vibrio* abundance with total length in juvenile angelsharks (*Squatina squatina*). Correlation between *Pseudoalteromonaceae* and *Vibrio sp*. abundance (gene copies/cm2) in skin *Squatina squatina* skin samples to total length (cm). Sampling date is represented by a colour gradient in the jitter plot. Shark ID of recaptured individuals are shown and data points connected by a solid grey line. Dashed line and grey areas represent confidence interval (CI), 95% for the fitted regression line.

### 3.4. Seasonality, zoning and mucus influence on *Squatina squatina* microbial diversity

Water and sediment samples exhibited a rather unchanged microbial community composition (S3 Fig) except for December 2023 when a high abundance of *Alteromonadaceae* (> 35%) was found in the water of Las Teresitas. Differences could not be attributed to changes in the water temperature or dissolved oxygen that remained unchanged (S2 Fig).

In relation to microbial load (gene copies), the abundances of *Pseudoalteromonadaceae* and *Vibrio* sp. were significantly higher in December compared to June in skin samples (Kruskal–Wallis test, p < 0.05), whereas no differences were found for oral and cloacal samples. According to microbial structure, zonation and time exerted interesting effects on the shark microbiota, specifically on that of the skin and mouth, with significant changes recorded at the Family level, not observed in cloaca samples (Fig 6, S9 Fig and S10 Fig). For most bacterial families, higher relative abundances were found in Zones 1 or 3, suggesting a zonation effect. In contrast, *Pseudoalteromonadaceae* and *Vibrio sp.* exhibited higher relative concentration in Zone 2 (the less exposed to open waters). Those differences could not be attributed to changes in the water or sediment microbiota (Kruskal–Wallis test, df = 2, p > 0.05). Time effects on skin were also evident, and higher relative abundances were generally recorded for summer samples except for *Pseudoaltermonadaceae* were higher relative values occurred in December 2023. Sampling date and transect zone interactions were only significant for *Rhodobacteraceae* and *Sphingomonadaceae* in skin samples (Scheirer-Ray-Hare test, p < 0.05).

**Fig 6:**
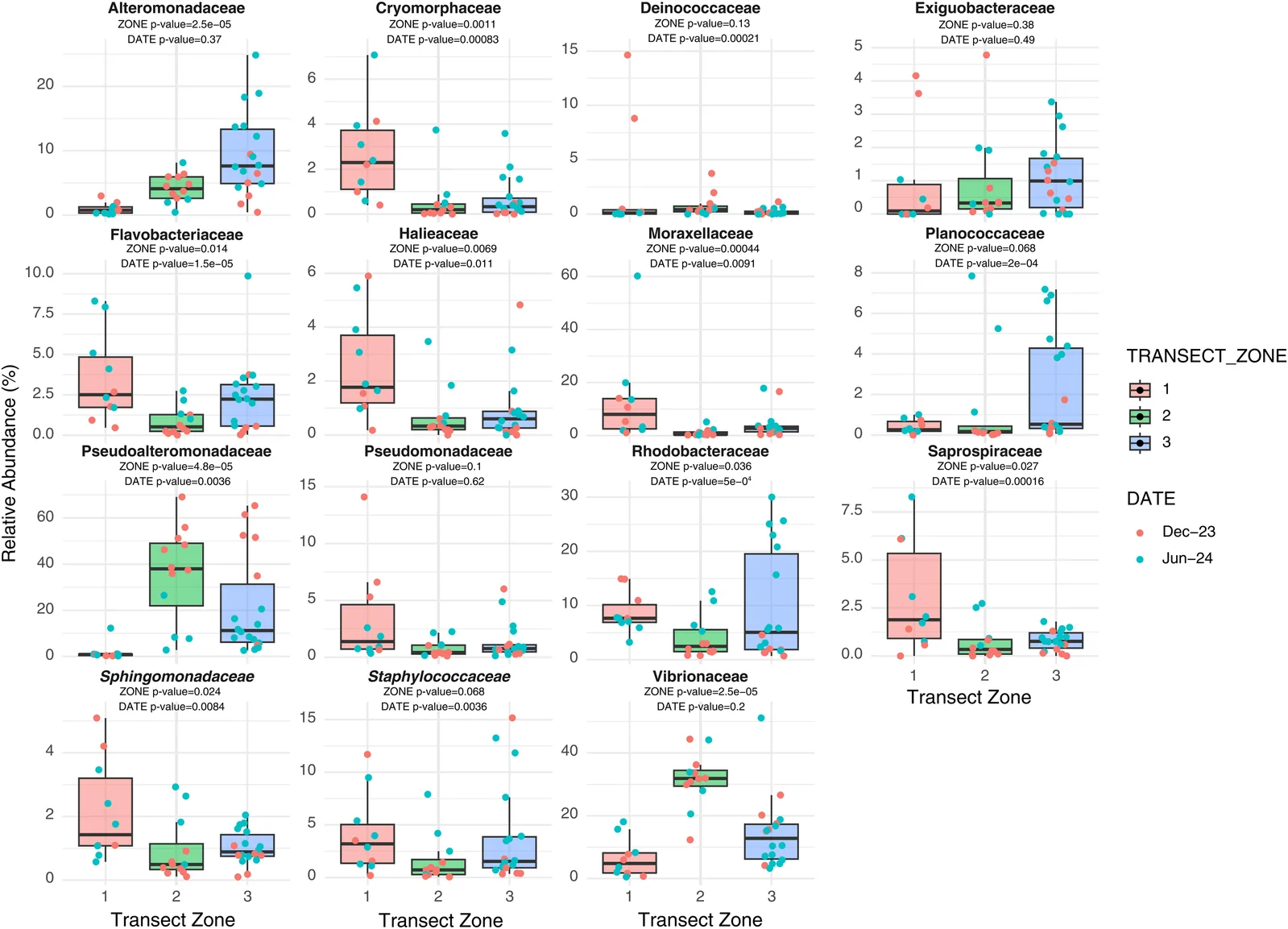
Differences in the relative abundance of dominant skin-associated bacterial families across transect zones and sampling dates. Box and whisker plot showing differences in relative abundances (%) of the 15 major identified families in the *Squatina squatina* skin microbiota according to transect zones in La Teresitas. Sampling dates are shown in different colours (December and June) is shown. Kruskal-Wallis Benjamini-Hochberg p-values for each selected bacterial Family according to DATE and ZONE are shown.

A high number of *S. squatina* specimens (n = 23) collected during June 2024 were classified according to the presence of mucus. Thirteen individuals exhibited slimy skin (high presence of mucus). The presence of mucus did not result in significant differences in alpha diversity indices (Kruskal–Wallis test, χ² = 3.131, df = 1, p = 0.077), despite changes in the relative abundance of some families were detected (S11 Fig).

## 4. Discussion

Shark skin microbiomes exhibit high species specificity [2]. Unlike teleost fishes, elasmobranchs display a phylosymbiosis pattern, indicating that their microbiomes are linked to host evolutionary history and traits [1]. Nevertheless, at first stages of development, the host microbiome should be more susceptible to changing environmental conditions. In this study, we provide the first insights into the early-life-stage microbial communities of the Critically Endangered angelshark and how they vary during its initial stages of development. Given the ethology of the species, both sand (sediment) and water were hypothesized to have an effect on the skin microbiome of the angelshark. As the length of individuals increases, the similarity of the water-skin and sediment-skin microbiome decreases, thus reflecting a higher specificity of the skin microbiome (Fig 7). Less strong correlations were found when oral or cloacal microbiomes were considered, suggesting a lesser influence of water and sediment on these body parts. In terms of microbial community members (number of shared ASVs), the surrounding sediment would be more influential since about 6% of phylotypes are shared between the skin and the sediment, in comparison to water (less than 1% shared ASVs). Similar results in terms of special richness were observed in the butterfly ray, *Gymnura altavela*, a species exhibiting also a preference for sandy bottoms, was also highly similar to the sediment composition [39]. In contrast, in large pelagic sharks such as *Carcharodon carcharias, Galeocerdo cuvier or Rhincodon typus*, external skin microbiomes are largely influenced by water microbes being the unique source of microbes for the host [5]. This is not a common rule since in other pelagic shark species little differences between the host microbiome of sharks and surrounding water have been reported in shared microbe species [12,40].

**Fig 7:**
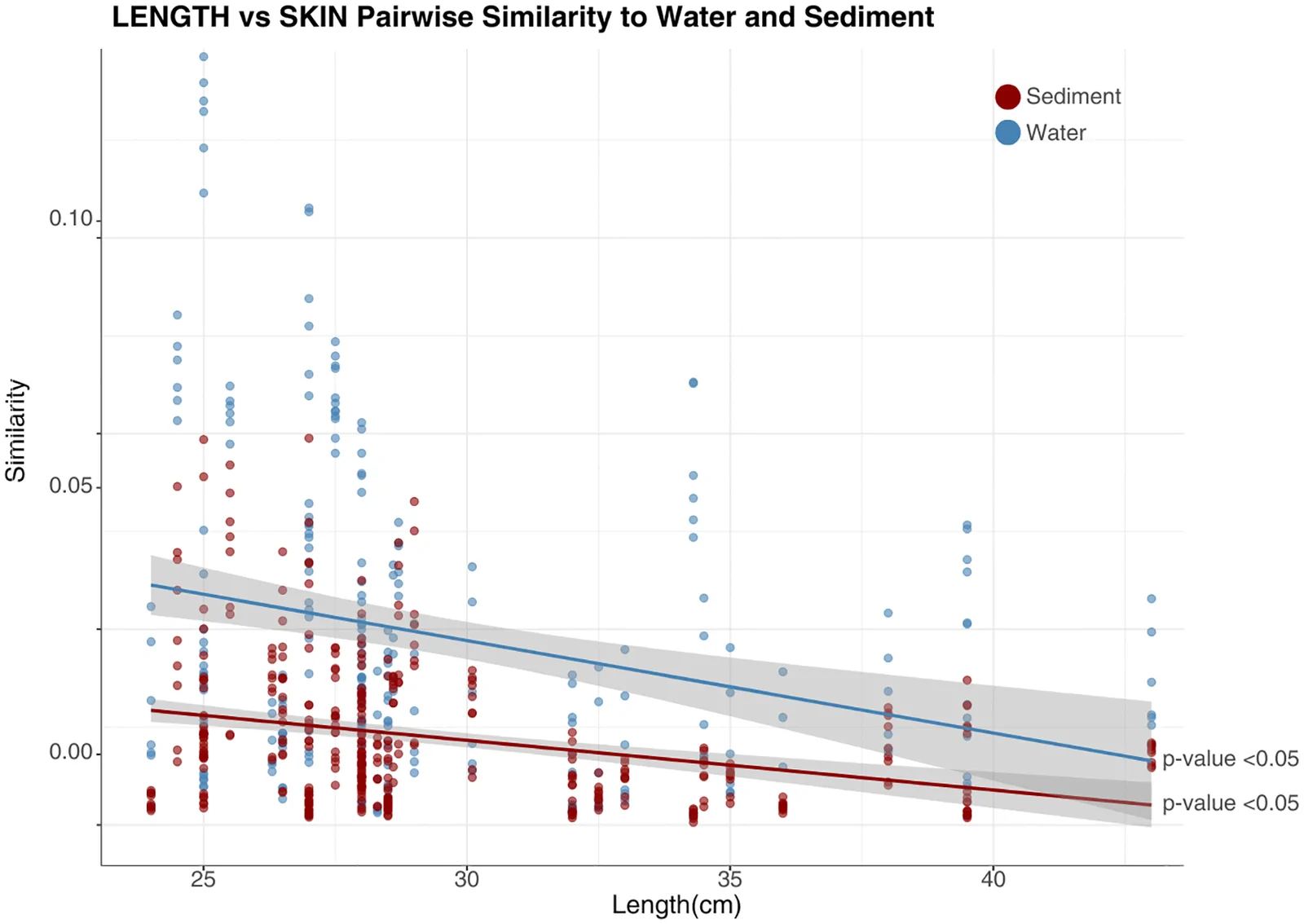
Correlation between skin–environment bacterial community similarity and total length of juvenile angelsharks (*Squatina squatina*). Pairwise similarity distance (Inverse Bray-Curtis) between Skin and Environmental samples (Water and Sediment) compared to *Squatina squatina* length (cm). Spearman correlation p-value represented for interaction. Linear model lines and grey areas represent confidence interval (CI), 95% for the fitted regression line.

In terms of differences in alpha diversity, it was clear that juvenile *S. squatina* microbiomes fall within the range of water samples, that is lower Shannon’s diversity and Richness indexes compared to sediment microbiomes. As expected, diversity indexes in shark skin microbiome were highly variable compared to oral or cloacal microbiomes, probably reflecting sediment heterogeneity, the life history of shark individuals, and the presence of skin mucus. In fact, in other shark species such as *Ginglymostoma cirratum*, *Negaprion brevirostris* and *Hypanus americanus* diversity of skin microbiomes was similar to that of the surrounding water and the presence of mucus had a homogenization effect among species [41]. Given our results showing a decrease in skin microbiome alpha diversity with increasing body size and the high homogeneity in other body parts, we suggest external skin microbiomes are subsequently modulated after environmental exposure. To reinforce the idea of the environment serving as a reservoir for new bacteria, the core microbiome analyses showed an almost exclusive presence of four relevant genera in shark body-parts that occur only sporadically in water or sediment samples (i.e., short period or individual samples).

A third analysis that can lead us to qualify the importance of water or sediment microorganisms in *S. squatina* is beta metrics. We chose Bray-Curtis (BC) dissimilarity, which summarizes differences between samples by considering sequence abundances [42,43], and compared (1/BC, similarity) between shark microbiomes, water and sediment samples pairwise (Fig 7, S12 Fig and S13 Fig). In early life stages (smaller sharks), water-to-skin similarity was higher than sediment-to-skin similarity, suggesting a stronger influence of water. During this early life stage, similarity between skin and the surrounding environment decreased significantly, showing that a specific microbial community is developed during early stages of growth in *S. squatina*. Interestingly, despite the burial behaviour of angelshark, sediment seemed to contribute less to the acquisition of new microbiome members. No such effects were observed for the cloacal and oral samples in the sample set used here, reinforcing the results observed from alpha diversity comparisons. As reported earlier, cloacal and oral microbiomes are more influenced by food intake [29] and in some instances, cloacal swabs have been used as proxies for the analysis of shark diet and gut microbiome [44,45].

To this point, it becomes interesting to gather information on what may be the drivers leading to changes in the body microbiomes of sharks during development and how do they eventually end up in a stable, balanced microbiome as suggested by Doane and collaborators for the whale shark [46]. In our work, we have identified at least two elements that modulate the external skin microbiome of the juvenile angelsharks. On the one hand, small-scale hydrodynamic differences, driven by the semi-enclosed construction of Las Teresitas beach, leads to three areas with a different exposure to open waters. We have shown that, at least at the family level, a zonation in shark microbiomes could be detected leading to a higher abundance of *Vibrio sp.* in the most isolated beach area (Zone 2). A previous study highlighted the presence of *Vibrio* sp. as a potential causative agent of meningitis in various shark species [47]. These results suggest that water quality may influence shark health, a critical issue for juvenile specimens. On the other hand, a time effect can cause a differentiation in shark microbiome. Our findings indicate that individuals sampled in December exhibited a distinct microbiome composition, characterized by a notable presence of *Pseudoalteromonadaceae, Vibrio sp*., and other described families. These differences may be related to individual size (i.e., growth), as the sharks sampled in December (within the scope of this study) were larger compared to those collected in June. In addition, no time differences could be attributed to sediment or to water samples, except for samples collected during December 2023. December 2023 was characterized by a heavy episode of African dust, “calima”, According to data available throughout the Gobierno de Canarias Net (Red de Control y Vigilancia de la Calidad del Aire en Canarias) at Piscina Municipal station (located 9,5 km from Las Teresitas Area), in December 2023 concentration of suspended particles in the air to be fourteen times higher compared to June 2023 (Gobierno de Canarias, s.f.). These episodes are known to cause sudden nutrient increases in the superficial water leading to blooms of opportunistic microbes [48,49]. In any case, these sporadic changes could be translated to the skin microbiome composition.

We have identified *Pseudoalteromonadaceae* as one of the most relevant components of the angelshark microbiome, reaching values up to 60% for some samples. *Pseudoalteromonas* sp. has been recognized as a key contributor to biofilm regulation, owing to its adhesion and inhibition capabilities, as reported in elasmobranch species [48,50,51]. In fact, we report that the abundance of *Pseudoalteromonadaceae* correlates with total shark length indicating an active recruitment of these bacteria and suggesting a protective role for juvenile angelsharks. In juvenile sea bass, *Pseudoalteromonadaceae* increased over time and with fish growth, and was closely associated with biofilm inhibition and pathogen control, contributing to improved bass fitness [52]. In shark species, no such relationship has been described; however, *Pseudoalteromonadaceae* has been observed as an important component of the core microbiome of *Carcharhinus melanopterus* and has been related to the recovery of healthy skin after injuries [21]. In addition, *Pseudoalteromonas* enriched biofilm layer could contribute to a defensive barrier against pathogens and play a role in regulating skin health during growth [1]. All these reports suggest an active role of this group as a protecting agent which may be relevant in early growth stages.

During the early stages of development, *S. squatina* microbiomes differentiate from the surrounding environment and specialize and help maintaining shark homeostasis. According to the IUCN guidelines, pollution and habitat degradation are among the most critical threats to elasmobranchs [53–55].Given the coastal habitat preference and benthic nature of juvenile angelsharks [15–17] anthropogenic influences such as wastewater runoff and climate change may promote the proliferation of pathogenic microbial taxa, placing early life stages at increased risk. Underscoring that any planned coastal engineering (e.g., new groynes, breakwaters, sediment replenishment) should undergo specific screening for likely microbiome shifts in *S. squatina* juveniles as part of impact assessment. Additionally, as observed by Pogoreutz [56], the similarities between bacterial communities in healthy and injured skin samples suggest that community structure may respond to exogenous environmental factors. Recent studies have shown that rising sea surface temperatures (SST) are altering the habitat use of adult angelsharks [17], which may, in turn, influence the structure and function of their skin-associated microbiomes. Genetic evidence in the Canary Islands *S. squatina* indicates Tenerife being one separate management unit, underscoring the importance of localized conservation [57].

From a conservation perspective, habitat management within conservation planning for critical or potential nursery areas of angelsharks should be regulated not only in terms of physical disturbance but also considering the environmental impacts on microbiomes. Assays on juvenile angelsharks could serve as an early-warning tool for monitoring and detecting direct environmental effects on individuals. This approach links angelshark conservation directly to coastal management decisions at the scale of a single bay and positions microbiome integrity as a measurable biological indicator for assessing short-term anthropogenic pressures.

## Supporting information

S1 Table Summary of environmental variables and sampling across sampling campaigns. Environmental data and number of angelshark individuals sampled and number of recaptures (in parenthesis) in each sampling campaign. Tidal conditions were categorized into three levels, low, medium, and high. Visibility was ranked in five categories, from 0 to 4, with 4 representing the highest water clarity.
(DOCX)

S2 Table Biological characteristics and sampling metadata of juvenile angelsharks (*Squatina squatina*). Study metadata including PIT tag identification number for each juvenile, total length (TL, cm), weight (g), sex (female, F; male, M), recapture status (“yes” if the individual was recaptured), transect zone where juveniles were found (Fig. 1), and mucus presence, recorded during the June 2024 campaign as present (“yes”) or absent (“no”).
(DOCX)

S3 Table Sequencing depth and ASV counts for samples included in microbiome analyses. Study reads and ASVs for each sample used. For posteriors analysis samples with low than 1000 reads were discarded.
(DOCX)

S1 Fig Procrustes analysis comparing Bray–Curtis NMDS configurations obtained from the non-rarefied and rarefied datasets. Procrustes analysis showing the concordance between Bray–Curtis NMDS configurations obtained from the non-rarefied and rarefied datasets (6,000 reads per sample).
(TIF)

S2 Fig Shared and unique bacterial ASVs among environmental samples and body sites of juvenile angelsharks (*Squatina squatina*). a) Venn diagram showing unique and shared ASVs between sample groups (Skin, Cloaca and Mouth of *Squatina squatina*, and Sediment and Water), ASV count and % represented b) UpSet plot with sample interactions, Intersection Size and Set Size represented. (TIF)

S3 Fig Relative abundance of bacterial orders across body sites of juvenile angelsharks (*Squatina squatina*) and environmental samples. Order-level stacked bar plots showing bacterial community composition of *Squatina squatina* tissue samples (Mouth, Cloaca and Skin) and environmental samples (Water and Sediment) collected from Las Teresitas.
(TIF)

S4 Fig Relative abundance of bacterial families across body sites of juvenile angelsharks (*Squatina squatina*) and environmental samples. Family-level stacked bar plots showing bacterial community composition of *Squatina squatina* tissue samples (Mouth, Cloaca and Skin) and environmental samples (Water and Sediment) collected from Las Teresitas.
(TIF)

S5 Fig Heatmap of core bacterial genera across body sites and environmental samples. Genus-level Heatmap showing core ASVs composition of *Squatina squatina* tissue samples (Mouth, Cloaca, and Skin) and environmental samples (Water and Sediment) collected from Las Teresitas at considered at relative abundances above 0.01 and minimum prevalences of 70%. ASV not characterized at Genus level were not included in the analysis. Prevalence shown as a blue-red gradient, being blue the lowest.
(TIF)

S6 Fig Contribution of bacterial families to between-group dissimilarity based on SIMPER analysis. SIMPER analysis showing the average contribution (%) of bacterial families to dissimilarity between each pair of sample locations.
(TIF)

S7 Fig Relationship between skin bacterial diversity and body weight in juvenile angelsharks (*Squatina squatina*). Scatter plots showing the correlation between Shannon diversity (left) and observed richness (right) index values in skin samples with *Squatina squatina* weight (g). Colour gradient of dots represents weight differences being darker the bigger individuals. Dashed line and grey areas represent confidence interval (CI), 95% for the fitted regression line.
(TIF)

S8 Fig Correlation of Shannon diversity and observed richness with total length in mouth and cloaca microbiomes of juvenile angelsharks (*Squatina squatina*). Scatter plots showing the correlation between Shanon diversity and observed Richness index values in mouth and cloaca samples with *Squatina squatina* Total Length (cm). Light orange dots corresponded to Mouth samples. Dark red dots are related to Cloaca samples. Colour gradient of dots represents weight differences being darker the heavier individuals. Dashed line and grey areas represent confidence interval (CI), 95% for the fitted regression line.
(TIF)

S9 Fig Differences in mouth microbiota composition across transect zones and sampling dates in juvenile angelsharks (*Squatina squatina*). Box and whisker plot showing differences in relative abundances (%) of the 15 major identified families in the *Squatina squatina* mouth microbiota according to transect zones in La Teresitas. Sampling dates are shown in different colours (December and June) is shown. Kruskal-Wallis Benjamini-Hochberg p-values for each selected bacterial Family according to DATE and ZONE are shown.
(TIF)

S10 Fig Spatial and temporal variation in cloaca-associated bacterial families of juvenile angelsharks (*Squatina squatina*). Box and whisker plot showing differences in relative abundances (%) of the 15 major identified families in the *Squatina squatina* cloaca microbiota according to transect zones in La Teresitas. Sampling dates are shown in different colours (December and June) is shown. Kruskal-Wallis Benjamini-Hochberg p-values for each selected bacterial Family according to DATE and ZONE are shown.
(TIF)

S11 Fig Differences in skin-associated bacterial families according to mucus presence in juvenile angelsharks (*Squatina squatina*). Box and whisker plots of the relative abundance (%) of the fifteen highest families according to the mucus presence in *Squatina squatina* skin microbiome. YES- slimy skin touch. NO- hard skin touch Kruskal-Wallis Benjamini-Hochberg p-values are shown for each comparison.
(TIF)

S12 Fig Correlation between mouth–environment microbial similarity and total length in juvenile angelsharks (*Squatina squatina*). Pairwise similarity distance (Inverse Bray-Curtis) between Mouth and Environmental samples (Water and Sediment) compared to *Squatina squatina* length (cm). Spearman correlation p-value represented for interaction. Linear model lines and grey areas represent confidence interval (CI), 95% for the fitted regression line.
(TIF)

S13 Fig Correlation between cloaca–environment bacterial similarity and total length in juvenile angelsharks (*Squatina squatina*). Pairwise similarity distance (Inverse Bray-Curtis) between Cloaca and Environmental samples (Water and Sediment) compared to *Squatina squatina* length (cm). Spearman correlation p-value represented for interaction. Linear model lines and grey areas represent confidence interval (CI), 95% for the fitted regression line.
(TIF)

## References

[1] Doane MP, Johnson CJ, Johri S, Kerr EN, Morris MM, Desantiago R, et al. The epidermal microbiome within an aggregation of leopard sharks (Triakis semifasciata) has taxonomic flexibility with gene functional stability across three time-points. Microb Ecol. 2023;85(2):747–64. doi: 10.1007/s00248-022-01969-y 35129649 PMC9957878

[2] Storo R, Easson C, Shivji M, Lopez JV. Microbiome analyses demonstrate specific communities within five shark species. Front Microbiol. 2021;12. doi: 10.3389/fmicb.2021.605285 PMC7904884 33643235

[3] Black C, Merly L, Hammerschlag N. Bacterial Communities in Multiple Tissues Across the Body Surface of Three Coastal Shark Species. Zool Stud. 2021;60:e69. doi: 10.6620/ZS.2021.60-69 35774257 PMC9168907

[4] Juste-Poinapen NMS, Yang L, Ferreira M, Poinapen J, Rico C. Community profiling of the intestinal microbial community of juvenile Hammerhead Sharks (Sphyrna lewini) from the Rewa Delta, Fiji. Sci Rep. 2019;9(1):7182. doi: 10.1038/s41598-019-43522-x 31073209 PMC6509141

[5] Pratte ZA, Perry C, Dove ADM, Hoopes LA, Ritchie KB, Hueter RE, et al. Microbiome structure in large pelagic sharks with distinct feeding ecologies. Anim Microbiome. 2022;4(1):17. doi: 10.1186/s42523-022-00168-x 35246276 PMC8895868

[6] Posadas N, Baquiran JIP, Nada MAL, Kelly M, Conaco C. Microbiome diversity and host immune functions influence survivorship of sponge holobionts under future ocean conditions. ISME J. 2022;16(1):58–67. doi: 10.1038/s41396-021-01050-5 34218251 PMC8692459

[7] Schmidt VT, Smith KF, Melvin DW, Amaral-Zettler LA. Community assembly of a euryhaline fish microbiome during salinity acclimation. Mol Ecol. 2015;24(10):2537–50. doi: 10.1111/mec.13177 25819646

[8] Lima LFO, Weissman M, Reed M, Papudeshi B, Alker AT, Morris MM, et al. Modeling of the coral microbiome: The influence of temperature and microbial network. mBio. 2020;11(2):e02691-19. doi: 10.1128/mBio.02691-19 32127450 PMC7064765

[9] Tarnecki AM, Burgos FA, Ray CL, Arias CR. Fish intestinal microbiome: Diversity and symbiosis unravelled by metagenomics. J Appl Microbiol. 2017;123(1):2–17. doi: 10.1111/jam.13415 28176435

[10] Doane MP, Haggerty JM, Kacev D, Papudeshi B, Dinsdale EA. The skin microbiome of the common thresher shark (Alopias vulpinus) has low taxonomic and gene function β-diversity. Environ Microbiol Rep. 2017;9(4):357–73. doi: 10.1111/1758-2229.12537 28418094

[11] Rosenberg E, Zilber-Rosenberg I. The hologenome concept of evolution after 10 years. Microbiome. 2018. doi: 10.1186/s40168-018-0457-9 PMC5922317 29695294

[12] Montemagno F, Romano C, Bastoni D, Cordone A, De Castro O, Stefanni S, et al. Shark microbiome analysis demonstrates unique microbial communities in two distinct mediterranean sea shark species. Microorganisms. 2024;12(3):557. doi: 10.3390/microorganisms12030557 38543607 PMC10975355

[13] Wilkins LGE, Leray M, O’Dea A, Yuen B, Peixoto RS, Pereira TJ, et al. Host-associated microbiomes drive structure and function of marine ecosystems. PLoS Biol. 2019;17(11):e3000533. doi: 10.1371/journal.pbio.3000533 31710600 PMC6874084

[14] Morey G, Barker J, Hood A. Squatina squatina, Angelshark. The IUCN red list of threatened species. http://www.iucnredlist.org. 2018.

[15] Jiménez-Alvarado D, Meyers EKM, Caro MB, Sealey MJ, Barker J. Investigation of juvenile angelshark (Squatina squatina) habitat in the Canary Islands with recommended measures for protection and management. Aquat Conserv. 2020;30(10). doi: 10.1002/aqc.3337

[16] Mead L, Jiménez Alvarado D, Meyers E, Barker J, Sealey M, Caro M, et al. Spatiotemporal distribution and sexual segregation in the Critically Endangered angelshark Squatina squatina in Spain’s largest marine reserve. Endang Species Res. 2023;51:233–48. doi: 10.3354/esr01255

[17] Meyers EKM, Tuya F, Barker J, Jiménez Alvarado D, Castro-Hernández JJ, Haroun R. Population structure, distribution and habitat use of the critically endangered angelshark, Squatina squatina, in the Canary Islands. Aquatic Conservation: Marine and Freshwater Ecosystems. 2017;27(6). doi: 10.1002/aqc.2769

[18] Chien H-W, Chen X-Y, Tsai W-P, Lee M. Inhibition of biofilm formation by rough shark skin-patterned surfaces. Colloids Surf B Biointerfaces. 2020;186:110738. doi: 10.1016/j.colsurfb.2019.110738 31869602

[19] Rostami S, Garipcan B. Evolution of antibacterial and antibiofouling properties of sharkskin-patterned surfaces. Surface Innovations. 2022;10(3):165–90. doi: 10.1680/jsuin.21.00055

[20] Vallespir Lowery N, Ursell T. Structured environments fundamentally alter dynamics and stability of ecological communities. Proc Natl Acad Sci U S A. 2019;116(2):379–88. doi: 10.1073/pnas.1811887116 30593565 PMC6329963

[21] Pogoreutz C, Gore M, Perna G, Ormond R, Clarke CR, Voolstra CR. Microenvironments of black-tip reef sharks (Carcharhinus melanopterus) provide niche habitats for distinct bacterial communities. Coral Reefs. 2024;44(1):145–62. doi: 10.1007/s00338-024-02593-7

[22] Givens CE, Ransom B, Bano N, Hollibaugh JT. Comparison of the gut microbiomes of 12 bony fish and 3 shark species. Mar Ecol Prog Ser. 2015;518. doi: 10.3354/meps11034

[23] Bahrndorff S, Alemu T, Alemneh T, Lund Nielsen J. The Microbiome of Animals: Implications for Conservation Biology. Int J Genomics. 2016;2016:5304028. doi: 10.1155/2016/5304028 27195280 PMC4852354

[24] Egerton S, Culloty S, Whooley J, Stanton C, Ross RP. The gut microbiota of marine fish. Front Microbiol. 2018;9:873. doi: 10.3389/fmicb.2018.00873 29780377 PMC5946678

[25] Perry CT, Pratte ZA, Clavere-Graciette A, Ritchie KB, Hueter RE, Newton AL, et al. Elasmobranch microbiomes: emerging patterns and implications for host health and ecology. Anim Microbiome. 2021;3(1):61. doi: 10.1186/s42523-021-00121-4 34526135 PMC8444439

[26] Smith HW. The retention and physiological role of urea in the Elasmobranchii. Biological Reviews. 1936;11(1):49–82. doi: 10.1111/j.1469-185x.1936.tb00497.x

[27] Lamont RJ, Koo H, Hajishengallis G. The oral microbiota: Dynamic communities and host interactions. Nat Rev Microbiol. 2018;16(12):745–59. doi: 10.1038/s41579-018-0089-x 30301974 PMC6278837

[28] McIntosh J, Greenhill A, Butcher P, Dewar M. Antimicrobial Resistant Pathogens in the Oral Cavity of White (Carcharodon carcharias), Bull (Carcharhinus leucas) and Tiger (Galeocerdo cuvier) Sharks from the East Coast of Australia. Curr Microbiol. 2025;82(7):300. doi: 10.1007/s00284-025-04272-4 40399711 PMC12095389

[29] Unger NR, Ritter E, Borrego R, Goodman J, Osiyemi OO. Antibiotic susceptibilities of bacteria isolated within the oral flora of Florida blacktip sharks: guidance for empiric antibiotic therapy. PLoS One. 2014;9(8):e104577. doi: 10.1371/journal.pone.0104577 25110948 PMC4128751

[30] Escánez Pérez A, Crespo Torres A, Rodríguez Ramallo S, Oñate M, Marrero Pérez J. Estudio del primer área de cría del angelote (Squatina squatina) descubierta en Canarias. Chronica naturae. 2016;62(6).

[31] Meyers EKM, Bañeras T, Barker J, Bousquet C, Jacoby DMP, Jimenez-Alvarado D, et al. Tagging and Tracking Toolkit for Angel Sharks. Angel Shark Project. 2026.

[32] Holm JB, Humphrys MS, Robinson CK, Settles ML, Ott S, Fu L, et al. Ultrahigh-Throughput Multiplexing and Sequencing of >500-Base-Pair Amplicon Regions on the Illumina HiSeq 2500 Platform. mSystems. 2019;4(1). doi: 10.1128/msystems.00029-19 PMC6381223 30801027

[33] López-Gutiérrez JC, Henry S, Hallet S, Martin-Laurent F, Catroux G, Philippot L. Quantification of a novel group of nitrate-reducing bacteria in the environment by real-time PCR. J Microbiol Methods. 2004;57(3):399–407. doi: 10.1016/j.mimet.2004.02.009 15134887

[34] Holmes AJ, Tujula NA, Holley M, Contos A, James JM, Rogers P, et al. Phylogenetic structure of unusual aquatic microbial formations in Nullarbor caves, Australia. Environ Microbiol. 2001;3(4):256–64. doi: 10.1046/j.1462-2920.2001.00187.x 11359511

[35] Thompson JR, Randa MA, Marcelino LA, Tomita-Mitchell A, Lim E, Polz MF. Diversity and dynamics of a north atlantic coastal Vibrio community. Appl Environ Microbiol. 2004;70(7):4103–10. doi: 10.1128/AEM.70.7.4103-4110.2004 15240289 PMC444776

[36] R Core Team. R: A language and environment for statistical computing. R Foundation for statiscal computing, Vienna, Austria. Open J Stat. 2023;13(02).

[37] Callahan BJ, McMurdie PJ, Rosen MJ, Han AW, Johnson AJA, Holmes SP. DADA2: High-resolution sample inference from Illumina amplicon data. Nat Methods. 2016;13(7):581–3. doi: 10.1038/nmeth.3869 27214047 PMC4927377

[38] McMurdie PJ, Holmes S. phyloseq: An R package for reproducible interactive analysis and graphics of microbiome census data. PLoS One. 2013;8(4):e61217. doi: 10.1371/journal.pone.0061217 23630581 PMC3632530

[39] Gonçalves E Silva F, Dos Santos HF, de Assis Leite DC, Lutfi DS, Vianna M, Rosado AS. Skin and stinger bacterial communities in two critically endangered rays from the South Atlantic in natural and aquarium settings. Microbiologyopen. 2020;9(12):e1141. doi: 10.1002/mbo3.1141 33226191 PMC7755814

[40] Karns RC, Cassandra R. Microbial community richness distinguishes shark species microbiomes in South Florida. https://nsuworks.nova.edu/occ_stuetd/453. 2017.

[41] Caballero S, Galeano AM, Lozano JD, Vives M. Description of the microbiota in epidermal mucus and skin of sharks (Ginglymostoma cirratum and Negaprion brevirostris) and one stingray (Hypanus americanus). PeerJ. 2020;8:e10240. doi: 10.7717/peerj.10240 33362953 PMC7747685

[42] Kers JG, Saccenti E. The power of microbiome studies: Some considerations on which alpha and beta metrics to use and how to report results. Front Microbiol. 2022;12:796025. doi: 10.3389/fmicb.2021.796025 35310396 PMC8928147

[43] Lozupone CA, Knight R. Global patterns in bacterial diversity. Proc Natl Acad Sci U S A. 2007;104(27):11436–40. doi: 10.1073/pnas.0611525104 17592124 PMC2040916

[44] van Zinnicq Bergmann MPM, Postaire BD, Gastrich K, Heithaus MR, Hoopes LA, Lyons K, et al. Elucidating shark diets with DNA metabarcoding from cloacal swabs. Mol Ecol Resour. 2021;21(4):1056–67. doi: 10.1111/1755-0998.13315 33527665

[45] Clark ZSR, Fish JJ, Butcher PA, Holland OJ, Sherman CDH, Rizzari J, et al. Insights into the diet and trophic ecology of white sharks (Carcharodon carcharias) gained through DNA metabarcoding analyses of cloacal swabs. Environmental DNA. 2023;5(6):1362–77. doi: 10.1002/edn3.454

[46] Doane MP, Reed MB, McKerral J, Farias Oliveira Lima L, Morris M, Goodman AZ, et al. Emergent community architecture despite distinct diversity in the global whale shark (Rhincodon typus) epidermal microbiome. Sci Rep. 2023;13(1):12747. doi: 10.1038/s41598-023-39184-5 37550406 PMC10406844

[47] Smith M, Warmolts D, Thoney D, Hueter R. The Elasmobranch Husbandry Manual: Captive Care of Sharks, Rays and Their Relatives. Ohio Biological Survey. 2004.

[48] Lührig K, Canbäck B, Paul CJ, Johansson T, Persson KM, Rådström P. Bacterial community analysis of drinking water biofilms in southern Sweden. Microbes Environ. 2015;30(1):99–107. doi: 10.1264/jsme2.ME14123 25739379 PMC4356470

[49] González-Toril E, Osuna S, Viúdez-Moreiras D, Navarro-Cid I, Toro S D del, Sor S. Impacts of Saharan dust intrusions on bacterial communities of the low troposphere. Scientific Reports. 2020;10(1). doi: 10.1038/s41598-020-63797-9 PMC7176723 32321958

[50] Holmström C, Kjelleberg S. Marine Pseudoalteromonas species are associated with higher organisms and produce biologically active extracellular agents. FEMS Microbiol Ecol. 1999;30(4):285–93. doi: 10.1111/j.1574-6941.1999.tb00656.x 10568837

[51] Goodman AZ, Papudeshi B, Mora M, Kerr EN, Torres M, Moffatt JN. Elasmobranchs exhibit species-specific epidermal microbiomes guided by denticle topography. bioRxiv. 2024.

[52] Rahmani A, Parizadeh L, Baud M, Francois Y, Bazire A, Rodrigues S, et al. Potential of marine strains of pseudoalteromonas to improve resistance of juvenile sea bass to pathogens and limit biofilm development. Probiotics Antimicrob Proteins. 2025;17(2):640–54. doi: 10.1007/s12602-023-10180-5 37851325

[53] Dulvy NK, Fowler SL, Musick JA, Cavanagh RD, Kyne PM, Harrison LR, et al. Extinction risk and conservation of the world’s sharks and rays. Elife. 2014;3:e00590. doi: 10.7554/eLife.00590 24448405 PMC3897121

[54] Dulvy NK, Pacoureau N, Rigby CL, Pollom RA, Jabado RW, Ebert DA, et al. Overfishing drives over one-third of all sharks and rays toward a global extinction crisis. Curr Biol. 2021;31(21):4773-4787.e8. doi: 10.1016/j.cub.2021.08.062 34492229

[55] Kyne PM, Jabado RW, Rigby CL, Dharmadi, Gore MA, Pollock CM. The thin edge of the wedge: extremely high extinction risk in wedgefishes and giant guitarfishes. Aquat Conserv. 2020;30(7). doi: 10.1002/aqc.3331

[56] Pogoreutz C, Gore MA, Perna G, Millar C, Nestler R, Ormond RF, et al. Similar bacterial communities on healthy and injured skin of black tip reef sharks. Anim Microbiome. 2019;1(1):9. doi: 10.1186/s42523-019-0011-5 33499949 PMC7807711

[57] Meyers EKM, Faure N, Jiménez-Alvarado D, Barker J, Toledo-Padilla H, Tuya F, et al. Distinct management units for the Critically Endangered angelshark (Squatina squatina) revealed in the Canary Islands. Conserv Genet. 2024;26(1):103–16. doi: 10.1007/s10592-024-01655-1

