# Supplementary material for: Microbiome plasticity during the early life-stage of the angelshark (Squatina squatina) in Las Teresitas (Canary Islands)
Source: PLoS One. 2026 Oct 28;21(10):e0361287. doi: 10.1371/journal.pone.0361287 (PMC13716170; doi:10.1371/journal.pone.0361287)
Supplement: Supplementary file 1 [file pone.0361287.s001.docx]

**Supplementary Table 1.** Environmental data, number of angelshark individuals sampled, and number of recaptures (in parentheses) for each sampling campaign. Tidal conditions were categorized into three levels, low, medium, and high. Visibility was ranked in five categories, from 0 to 4, with 4 representing the highest water clarity. Enviromental sample designated for microbiome samples analysed in each campaign.

| Data | Sampling zone | Number of individuals sampled (recaptures) | Tide | Visibility | Temperature (ºC) | Dissolved Oxygen  (ppm)  (DO %) | Salinity (ppt) | Enviromental sample |
| --- | --- | --- | --- | --- | --- | --- | --- | --- |
| December 2023 | 1 | 4 | Low | 4 | 22.10 | 6.40 (93.40) | 37.00 | Sediment/Water |
|  | 2 | 8 | Low | 4 | 21.90 | 6.32 (91.50) | 36.00 | - |
|  | 3 | 6 | Low | 4 | 22.10 | 6.32 (93.40) | 37.00 | Sediment/Water |
| March 2024 | 1 | 0 | Med | 2 | 20.20 | 6.59 (90.10) | 36.32 | Sediment |
|  | 2 | 1 (1) | Med | 3 | 20.00 | 6.49 (88.40) | 36.50 | Sediment/Water |
|  | 3 | 0 | Med | 3 | 20.00 | 6.49 (88.40) | 36.50 | Water |
| June 2024 | 1 | 6 | Med | 3 | 21.90 | 6.32 (88.70) | 35.66 | Sediment |
|  | 2 | 4 | Med | 3 | 21.90 | 6.32 (88.70) | 35.66 | Sediment/Water |
|  | 3 | 14 (2) | Med | 3 | 21.90 | 6.32 (88.70) | 35.66 | Sediment/Water |
| December 2024 | 1 | 0 | Med | 2 | 21.20 | 6.49 (90.30) | 35.66 | - |
|  | 2 | 0 | Med | 2 | 21.40 | 6.57 (91.20) | 36.59 | - |
|  | 3 | 2 (2) | Med | 2 | 21.49 | 6.49 (90.50) | 36.67 | - |
