# Supplementary material for: Microbiome plasticity during the early life-stage of the angelshark (Squatina squatina) in Las Teresitas (Canary Islands)
Source: PLoS One. 2026 Oct 28;21(10):e0361287. doi: 10.1371/journal.pone.0361287 (PMC13716170; doi:10.1371/journal.pone.0361287)
Supplement: Supplementary file 2 [file pone.0361287.s002.docx]

| PIT tag | DATE | TL (cm) | WEIGHT (g) | SEX | TRANSECT ZONE | MUCUS | Shark body parts sampled |
| --- | --- | --- | --- | --- | --- | --- | --- |
| 45440 | 06/29/2024 | 29 | 205 | M | 3 | No | Skin/Mouth |
| 45448 | 06/29/2024 | 28.5 | 200 | F | 3 | No | Skin/Mouth |
| 45450 | 06/28/2024 | 27 | 159 | M | 2 | No | Skin/Mouth |
| 45454 | 06/29/2024 | 28 | 215 | M | 3 | Yes | Skin/Mouth |
| 45456 | 06/27/2024 | 25 | 170 | M | 1 | Yes | Skin/Mouth |
| 45459 | 06/28/2024 | 27.5 | 188 | M | 2 | Yes | Skin/Mouth |
| 45460 | 12/14/2023 | 36 | 427 | F | 3 | No | Skin/Mouth/Cloaca |
| 45461 | 06/29/2024 | 28 | 206 | F | 3 | No | Skin/Mouth |
| 45462 | 12/13/2023 | 32 | 371 | M | 3 | No | Skin/Mouth/Cloaca |
| 45462 | 06/29/2024 | 40 | 612 | M | 3 | No | Skin/Mouth |
| 45464 | 06/27/2024 | 28.7 | 184 | M | 1 | Yes | Skin/Mouth |
| 45465 | 12/13/2023 | 32.5 | 352 | F | 2 | No | Skin/Mouth/Cloaca |
| 45467 | 06/27/2024 | 24.5 | 145 | F | 1 | Yes | Skin/Mouth |
| 45470 | 12/14/2023 | 28.3 | 214 | M | 3 | No | Skin/Mouth/Cloaca |
| 45472 | 06/29/2024 | 28 | 188 | M | 3 | Yes | Skin/Mouth |
| 45477 | 06/29/2024 | 28 | 198 | M | 3 | No | Skin/Mouth |
| 45478 | 12/14/2023 | 28 | 215 | F | 3 | No | Skin/Mouth/Cloaca |
| 45480 | 12/13/2024 | 34 | 359 | M | 3 | No | Skin/Mouth |
| 45480 | 06/29/2024 | 27.5 | 184 | M | 3 | Yes | Skin/Mouth |
| 45483 | 12/13/2023 | 28.5 | 232 | F | 2 | No | Skin/Mouth/Cloaca |
| 45486 | 12/14/2023 | 33 | 388 | M | 3 | No | Skin/Mouth/Cloaca |
| 45487 | 12/13/2023 | 28 | 205 | M | 2 | No | Skin/Cloaca |
| 45489 | 12/14/2023 | 28.5 | 230 | M | 3 | No | Skin/Mouth/Cloaca |
| 45490 | 06/29/2024 | 26.5 | 160 | F | 3 | Yes | Skin/Mouth |
| 45501 | 06/27/2024 | 27 | 180 | M | 1 | Yes | Skin/Mouth |
| 45504 | 12/12/2023 | 28.6 | 185 | M | 1 | No | Skin/Cloaca |
| 45506 | 06/29/2024 | 27 | 195 | F | 3 | No | Skin/Mouth |
| 45512 | 12/13/2023 | 30.1 | 274 | F | 2 | No | Skin/Mouth/Cloaca |
| 45513 | 12/14/2023 | 32 | 314 | M | 3 | No | Skin/Mouth/Cloaca |
| 45513 | 06/29/2024 | 38 | 480 | M | 3 | No | Skin/Cloaca |
| 45513 | 12/13/2024 | 43 | 703 | M | 3 | No | Skin/Mouth |
| 45518 | 06/29/2024 | 25 | 144 | F | 3 | Yes | Skin/Mouth |
| 45520 | 06/29/2024 | 28 | 188 | M | 3 | No | Skin/Mouth |
| 45521 | 12/13/2023 | 24 | 245 | F | 2 | No | Skin/Mouth/Cloaca |
| 45527 | 06/29/2024 | 25 | 121 | M | 3 | No | Skin/Mouth |
| 45528 | 12/13/2023 | 34.5 | 377 | F | 2 | No | Skin/Mouth/Cloaca |
| 45528 | 03/21/2024 | 39.5 | 480 | F | 2 | No | Skin/Mouth/Cloaca |
| 45532 | 12/13/2023 | 35 | 401 | F | 2 | No | Skin/Mouth/Cloaca |
| 45543 | 12/12/2023 | 34.3 | 339 | F | 1 | No | Skin/Mouth |
| 45566 | 06/28/2024 | 28 | 214 | F | 2 | Yes | Skin/Mouth |
| 45599 | 12/12/2023 | 26.3 | 153 | F | 1 | No | Skin/Mouth |
| 45603 | 12/12/2023 | 27 | 170 | M | 1 | No | Skin/Mouth |
| 45623 | 06/27/2024 | 25.5 | 146 | F | 1 | Yes | Skin/Mouth |
| 45624 | 06/28/2024 | 25 | 132 | F | 2 | No | Skin/Mouth |
| 45638 | 06/27/2024 | 26.5 | 149 | F | 1 | Yes | Skin/Mouth |

Supplementary Table 2. Study metadata including PIT tag identification number for each juvenile, total length (TL, cm), weight (g), sex (female, F; male, M), recapture status ("yes" if the individual was recaptured), transect zone where juveniles were found (Fig. 1), and mucus presence, recorded during the June 2024 campaign as present ("yes") or absent ("no").
