# Supplementary material for: Microbiome plasticity during the early life-stage of the angelshark (Squatina squatina) in Las Teresitas (Canary Islands)
Source: PLoS One. 2026 Oct 28;21(10):e0361287. doi: 10.1371/journal.pone.0361287 (PMC13716170; doi:10.1371/journal.pone.0361287)
Supplement: Supplementary file 3 [file pone.0361287.s003.docx]

| SampleID | Reads | ASVs |
| --- | --- | --- |
| 45440JUN24M | 47022 | 318 |
| 45440JUN24S | 27794 | 651 |
| 45448JUN24M | 34666 | 230 |
| 45448JUN24S | 22588 | 424 |
| 45450JUN24M | 2913 | 45 |
| 45450JUN24S | 8622 | 123 |
| 45454JUN24M | 36371 | 127 |
| 45454JUN24S | 8481 | 262 |
| 45456JUN24M | 15870 | 213 |
| 45456JUN24S | 18890 | 379 |
| 45459JUN24M | 38718 | 138 |
| 45459JUN24S | 23676 | 490 |
| 45460DEC23C | 44841 | 210 |
| 45460DEC23M | 55980 | 469 |
| 45460DEC23S | 55318 | 205 |
| 45461JUN24M | 35386 | 148 |
| 45461JUN24S | 55995 | 1047 |
| 45462DEC23C | 36136 | 45 |
| 45462DEC23M | 39218 | 222 |
| 45462DEC23S | 39806 | 198 |
| 45464JUN24M | 19786 | 188 |
| 45464JUN24S | 15080 | 459 |
| 45465DEC23C | 18777 | 87 |
| 45465DEC23M | 33240 | 113 |
| 45465DEC23S | 52602 | 263 |
| 45467JUN24M | 13718 | 152 |
| 45467JUN24S | 33008 | 666 |
| 45470DEC23C | 10951 | 68 |
| 45470DEC23M | 55866 | 337 |
| 45470DEC23S | 20124 | 234 |
| 45472JUN24M | 29580 | 248 |
| 45472JUN24S | 26550 | 447 |
| 45477JUN24M | 44760 | 125 |
| 45477JUN24S | 29637 | 210 |
| 45478DEC23C | 16431 | 139 |
| 45478DEC23M | 56709 | 218 |
| 45478DEC23S | 13606 | 218 |
| 45480DEC24M | 3745 | 53 |
| **45480DEC24S** | **983** | **50** |
| 45480JUN24M | 45260 | 342 |
| 45480JUN24S | 28910 | 502 |
| 45483DEC23C | 11938 | 85 |
| 45483DEC23M | 43375 | 187 |
| 45483DEC23S | 34353 | 196 |
| 45486DEC23C | 40636 | 83 |
| 45486DEC23M | 33416 | 138 |
| 45486DEC23S | 44205 | 293 |
| 45487DEC23C | 21638 | 116 |
| 45487DEC23S | 55680 | 344 |
| 45489DEC23C | 6000 | 67 |
| 45489DEC23M | 19843 | 117 |
| 45489DEC23S | 31959 | 160 |
| 45490JUN24M | 51952 | 196 |
| 45490JUN24S | 38570 | 584 |
| 45501JUN24M | 18346 | 173 |
| 45501JUN24S | 15693 | 381 |
| 45504DEC23C | 12193 | 143 |
| 45504DEC23S | 50136 | 678 |
| 45506JUN24M | 48142 | 120 |
| 45506JUN24S | 8327 | 125 |
| 45512DEC23C | 6782 | 76 |
| 45512DEC23M | 6918 | 265 |
| 45512DEC23S | 66374 | 915 |
| 45513DEC23C | 8066 | 80 |
| 45513DEC23M | 56179 | 232 |
| 45513DEC23S | 30467 | 162 |
| 45513DEC24M | 10686 | 89 |
| 45513DEC24S | 9032 | 160 |
| 45513JUN24C | 9331 | 96 |
| 45513JUN24S | 11903 | 252 |
| 45518JUN24M | 19047 | 137 |
| 45518JUN24S | 31513 | 644 |
| 45520JUN24M | 27358 | 100 |
| 45520JUN24S | 8470 | 199 |
| 45521DEC23C | 12058 | 136 |
| 45521DEC23M | 41484 | 282 |
| 45521DEC23S | 42656 | 204 |
| 45527JUN24M | 24890 | 216 |
| 45527JUN24S | 39977 | 537 |
| 45528DEC23C | 14343 | 160 |
| 45528DEC23M | 38185 | 179 |
| 45528DEC23S | 55669 | 430 |
| 45528MAR24C | 28672 | 549 |
| 45528MAR24M | 13590 | 158 |
| 45528MAR24S | 22047 | 127 |
| 45532DEC23C | 21892 | 141 |
| 45532DEC23M | 61683 | 263 |
| 45532DEC23S | 54991 | 310 |
| **45543DEC23M** | **18** | **2** |
| 45543DEC23S | 9968 | 122 |
| 45566JUN24M | 28625 | 129 |
| 45566JUN24S | 7170 | 180 |
| 45599DEC23M | 70565 | 1056 |
| 45599DEC23S | 113056 | 1142 |
| 45603DEC23M | 22055 | 194 |
| 45603DEC23S | 33762 | 973 |
| 45623JUN24M | 14688 | 176 |
| 45623JUN24S | 37460 | 1036 |
| 45624JUN24M | 47319 | 256 |
| 45624JUN24S | 50523 | 631 |
| 45638JUN24M | 11726 | 203 |
| 45638JUN24S | 14264 | 251 |
| SedimentDEC231R1 | 53243 | 1413 |
| SedimentDEC231R3 | 75979 | 1755 |
| SedimentDEC232R1 | 112459 | 2168 |
| SedimentDEC232R3 | 50917 | 1504 |
| SedimentJUN241R1 | 102537 | 2462 |
| SedimentJUN242R1 | 92960 | 1721 |
| SedimentJUN243R1 | 98629 | 1872 |
| SedimentMAR242R1 | 92219 | 1983 |
| SedimentMAR242R2 | 87021 | 1914 |
| WaterDEC23R1 | 57233 | 286 |
| WaterDEC23R3 | 60964 | 303 |
| WaterJUN24R2 | 52541 | 366 |
| WaterJUN24R3 | 65694 | 268 |
| WaterMAR24R2 | 62713 | 311 |
| WaterMAR24R3 | 63538 | 347 |
